# Occupational noise exposure and cardiovascular disease risk factors in China: mediating role of hearing loss

**DOI:** 10.3389/fpubh.2026.1901898

**Published:** 2026-08-04

**Authors:** Qiyou Tan, Yong Hu, Panqi Xue, Fei Li, Fang Wei, Hongwei Xie, Zhen Zhou, Changjian Quan, Dongming Wang, Hua Zou, Xiaoming Lou, Lifang Zhou

**Affiliations:** 1 Department of Occupational Health and Radiation Protection, Zhejiang Provincial Center for Disease Control and Prevention, Hangzhou, China; 2 Department of Occupational & Environmental Health, School of Public Health, Tongji Medical College, Huazhong University of Science and Technology, Wuhan, China

**Keywords:** blood glucose, blood pressure, electrocardiogram abnormality, hearing loss, occupational noise

## Abstract

**Background:**

The health risks of noise pollution have attracted global attention, especially the potential cardiovascular effects of occupational noise exposure. Our study aimed to investigate the associations of occupational noise with cardiovascular disease risk factors [fasting blood glucose (FBG), systolic blood pressure (SBP), diastolic blood pressure (DBP), and electrocardiogram (ECG) abnormality], and further evaluate the possible mediating role of hearing loss in the associations.

**Methods:**

This cross-sectional study included 64,382 participants exposed to occupational noise in China. Individual level of noise exposure with an 8-h equivalent A-sound level (L_Aeq, 8h_) was measured by personal noise dosimeter. And the noise exposure level of workers was approximated using the average of measured individual noise values within each enterprise. Generalized linear models and logistic regression models were used to analyze the associations of noise exposure with cardiovascular disease risk factors. Mediation analyses were performed to assess the mediating roles of hearing loss on above associations.

**Results:**

L_Aeq, 8h_ was positively associated with FBG (β = 0.006, 95% CI: 0.001–0.011), SBP (β = 0.036, 95% CI: 0.003–0.068), and risk of ECG abnormality (OR = 1.014, 95% CI: 1.009–1.019). We observed positive linear dose-response associations of noise exposure with blood pressure, as well as non-linear relationships with FBG and ECG abnormality risk [L_Aeq, 8h_ exceeded around 82 dB(A) as a turning point]. Age, dust exposure, high temperature, and length of service significantly modified above associations (*P* modification < 0.05), and the effects were more obvious in participants >43 years, workers with dust or high temperature exposure, and workers with length of service >5 years. Hearing loss mediated 3.7%−10.9% of the associations of noise exposure with elevated blood pressure and risk of ECG abnormality.

**Conclusions:**

Occupational noise exposure was associated with adverse changes in FBG, blood pressure, and risk of ECG abnormality. Older people, workers exposed to dust or high temperature, and those with longer working duration were more susceptible. Hearing loss might play potential role in cardiovascular damage induced by noise exposure. Efficient strategies for reducing occupational noise exposure should be enhanced to control the cardiovascular threat from occupational noise pollution.

## 1 Introduction

Noise, a persistent environmental problem which defined as “unwanted sound”, is ubiquity distributed in various scenes of human life (1). Anthropogenic activities, including industrial manufacturing, road traffic, aircraft operations, and recreational activities like singing and dancing, have contributed to a rapid and sustained elevation of environmental noise levels in recent years (2). According to the World Health Organization, about 40% of the European population are exposed to noise levels exceeding 55 A-weight decibels [dB(A)] (3). Moreover, noise exposure has resulted in the loss of approximately 1.6 million healthy life-years in Europe (4). Notably, noise exposure is particularly serious in the workplace (5). Previous studies have reported that 22 million workers in the United States are exposed to high levels of occupational noise, and about 20% of laborers in Europe and Australia are affected by occupational noise exposure (6, 7). As a major industrial manufacturing country, China has 25.1 % of manufacturing employees working in environments with excessive noise levels (8).

Noise exposure can induce a wide range of adverse health effects, including both auditory and non-auditory health outcomes (9). Noise pollution and its associated health impacts, especially metabolic disorders and cardiovascular damage, have emerged as major global public health concerns (10). Accumulating epidemiological evidences have suggested a significant association between noise exposure and harmful cardiovascular effects in populations from the United Kingdom, Switzerland, China and so on (11–14). These findings indicated that noise exposure may contribute to the development of cardiovascular diseases (15). It is hypothesized that chronic noise exposure disrupts autonomic and endocrine system regulation, leading to detrimental alterations in cardiovascular disease risk factors such as blood glucose and blood pressure (16). However, the adverse effect of high-intensity occupational noise exposure on cardiovascular health remains insufficiently investigated, particularly in China, one of the world's largest industrial manufacturing countries.

Sustained noise exposure directly affects the auditory system, causing alterations in hearing thresholds that may ultimately progress to hearing loss (17). Meanwhile, hearing loss has been proposed to be related with cardiovascular disease. A cross-sectional study from the National Health and Nutrition Examination Survey (NHANES) found that bilateral high-frequency hearing loss was positively associated with the presence of coronary heart disease after adjustment for potential covariates (18). Another study conducted among aircraft-manufacturing workers suggested that participants with high and median hearing loss had higher risk of hypertension relative to workers with low hearing loss (19). Based on above evidences, we hypothesized that hearing loss might contribute to the detrimental cardiovascular effects induced by occupational noise exposure.

We conducted this study among 64,382 workers exposed to occupational noise, and the objectives were: to investigate the associations of occupational noise exposure and hearing loss with cardiovascular disease risk factors; and to evaluate the potential mediating role of hearing loss in the relationships between occupational noise exposure and cardiovascular disease risk factors.

## 2 Methods

### 2.1 Study population

A total of 64,382 individuals aged 18–76 years working with noise exposure in Zhejiang province, China were obtained from the Key Occupational Disease Surveillance Project in 2024 with a cross-sectional survey. This survey was implemented to assess the health status of occupational population exposed to noise. Meanwhile, individual-level data was collected through questionnaires, professional physical examinations, and blood samples measurements. Written informed consent was signed by all participants. This study was approved by the Ethics Committee of the Zhejiang Provincial Center for Disease Control and Prevention (2022-026-01).

### 2.2 Noise exposure assessment

Individual level of noise exposure with an 8-h equivalent A-sound level (L_Aeq, 8h_) was determined by personal noise dosimeter (dBagde2, Casella, UK or QUEST Noisepro, 3M, US) according to the Chinese national occupational hygiene standard (GBZ/T 189.8-2007 Measurement of Physical Agents in Workplace Part 8: Noise). The noise exposure level of the noise-exposed workers was expressed as the average of all individual noise measurements within the employment enterprise. Subjects exposed to noise with an L_Aeq, 8h_ ≥80 dB(A) (sound level that harmful to health) were defined as noise-exposed employees (20). In the current study, noise-exposed subjects were classified into low-exposed and high-exposed groups according to the median value of L_Aeq, 8h_ [84.15 dB(A)].

### 2.3 Assessment of hearing loss

In accordance with the protocol of Chinese national standard of pure-tone air and bone conduction threshold audiometry (GB/T 16296.1–2018), the pure-tone audiometry examination was performed by professional technicians in a sound-isolating room. Subject's binaural pure-tone air conduction thresholds at 0.5, 1, 2, 3, 4, and 6 kHz were measured using a normalized pure-tone audiometer with intensity range from −10 to 120 dB. Binaural high-frequency threshold on average (BHFTA) was calculated as binaural pure-tone average of hearing thresholds at 3, 4, and 6 kHz. And binaural speech-frequency threshold on average (BSFTA) was calculated as worse ear pure-tone average of hearing thresholds at 0.5, 1, 2, and 4 kHz. High-frequency hearing loss (HFHL) and speech-frequency hearing loss (SFHL) were defined as BHFTA more than 40 dB and BSFTA more than 25 dB, respectively (21, 22).

### 2.4 Outcomes

The primary health outcomes of this study were fasting blood glucose (FBG), blood pressure, and electrocardiogram (ECG) abnormality. Fasting blood samples were collected by health care professionals, and the concentrations of FBG were measured using an automatic biochemical analyzer in clinical laboratories. Blood pressure including systolic blood pressure (SBP) and diastolic blood pressure (DBP) on the right arm were measured through a digital sphygmomanometer with a sitting position after 5 min of rest. Two times of blood pressure measurements were adopted and the mean value was recorded. Before the electrocardiogram examination, subjects rested in a supine position with 5 min. Then, the ECG examination was operated by a professional physician and measured using an electrocardiograph with conventional 12-lead electrocardiograms in a supine position. ECG results were interpreted according to the international standard of ECG and were coded on the basis of the Minnesota Code (23). And the results were regarded as abnormal except for normal electrocardiogram and sinus rhythm, including Q-QS wave abnormalities, left ventricular hypertrophy, complete bundle branch block or intraventricular conduction delay, ST-T segment changes, and so on.

### 2.5 Statistical analyses

Descriptive analyses were performed for covariates, noise exposure, hearing loss, and health outcomes in all participants. Categorical and continuous variables were described as number (percentage) and mean ± standard deviation (SD), respectively. Comparisons in general characteristics across noise-exposed and non-exposed groups were conducted through Student's *t*-test for continuous variables and chi-square test for categorical variables.

Generalized linear models were used to analyze the associations of noise exposure with FBG and blood pressure. Logistic regression models were used to assess the associations of noise exposure with risk of ECG abnormality and hearing loss. We also conducted categorical analyses with non-exposed participants [L_Aeq, 8h_ < 80 dB(A)] as reference group, and noise-exposed participants were categorized into low-exposed and high-exposed groups based on the median of L_Aeq, 8h_ [84.15 dB(A)]. The dose-dependent relationships of L_Aeq, 8h_ with FBG, blood pressure, and risk of ECG abnormality were graphically depicted by restricted cubic spline (RCS) models with 3 knots (the 25th, 50th, and 75th percentile of L_Aeq, 8h_). All models were adjusted for potential confounders including age, gender, height, weight, smoking status, dust exposure, high temperature exposure, hearing protection usage, and length of service. In addition, stratified analyses were conducted by several characteristics (age, gender, BMI, dust exposure, high temperature exposure, and length of service), and a product term of stratified characteristic with noise exposure was added in models to estimate the effect modification.

Moreover, we evaluated the associations of categorical noise exposure with BHFTA and BSFTA using generalized linear models. *P* for trend was then examined through including the median L_Aeq, 8h_ of each noise exposure group as a continuous variable within the statistical models. The models concerning associations of BHFTA and BSFTA with FBG, blood pressure, and ECG abnormality were as follows, Model 1: crude model; Model 2: adjusted for age, gender, height, weight, and smoking status; Model 3: Model 2 further adjusted for dust exposure, high temperature exposure, hearing protection usage, and length of service. Mediation analyses were performed to assess the potential mediating roles of BHFTA and BSFTA on the associations of noise exposure with cardiovascular disease risk factors. The total effect of occupational noise exposure on cardiovascular disease risk factors was composed by mediated effects (the effect explained by BHFTA or BSFTA) and direct effect. We conducted mediation analyses using the *mediation* R package which could compute mediated effects and mediating proportion.

All statistical analyses were conducted in R version 4.1.2 (R Foundation for Statistical Computing, Austria). Statistical significance was defined as *P* < 0.05 (two-sided).

## 3 Results

### 3.1 Characteristics of study population

In the present study, the mean age of all 64,382 participants (51,056 noise-exposed and 13,326 non-exposed) was 43.27 years and 83.23% of them were males (Table 1). The mean height and weight were 166.81 cm and 67.44 kg, respectively. Compared to non-exposed participants, noise-exposed participants exhibited older age, more proportion of hearing protection usage, less males, dust exposure, and high temperature exposure, and lower height, weight, and length of service. Noteworthily, noise-exposed subjects had more proportion of high-frequency and speech-frequency hearing loss, and higher levels of SBP and DBP (all *P* < 0.05).

**Table 1:** General and health characteristics of participants.

General characteristics	Total	Non-exposed	Noise-exposed	*P*-value
Participants (*N*)	64,382	13,326	51,056	–
Male, *n* (%)	53,583 (83.23)	11,305 (84.83)	42,278 (82.81)	< 0.001
Age, years, mean ± SD	43.27 ± 10.61	42.90 ± 10.74	43.37 ± 10.58	< 0.001
Height, cm, mean ± SD, (*n* = 49,684)	166.81 ± 7.43	167.46 ± 7.41	166.62 ± 7.42	< 0.001
Weight, kg, mean ± SD, (*n* = 49,826)	67.44 ± 11.56	68.05 ± 11.67	67.26 ± 11.52	< 0.001
Smokers, *n* (%)	44,635 (69.33)	41,17 (30.89)	15,630 (30.61)	0.531
Hearing protection usage, *n* (%)	61,679 (95.80)	12,557 (94.23)	49,122 (96.21)	< 0.001
Length of service, years, mean ± SD	5.89 ± 5.93	6.59 ± 6.40	5.71 ± 5.79	< 0.001
Dust exposure, *n* (%)	33,146 (51.48)	7,097 (53.26)	26,049 (51.02)	< 0.001
High temperature exposure, *n* (%)	8,345 (12.96)	2,988 (22.42)	5,357 (10.49)	< 0.001
L_Aeq, 8h_, dB(A), mean ± SD	83.11 ± 4.17	77.25 ± 2.26	84.64 ± 3.05	< 0.001
Hearing loss				
High-frequency hearing loss, *n* (%)	7,925 (12.31)	1,378 (10.34)	6,547 (12.82)	< 0.001
Speech-frequency hearing loss, *n* (%)	1,6512 (25.65)	2,985 (22.40)	13,527 (26.49)	< 0.001
Health outcomes				
FBG, mmol/L, mean ± SD (*n* = 14,375)	5.30 ± 1.33	5.30 ± 1.29	5.31 ± 1.34	0.648
SBP, mmHg, mean ± SD (*n* = 64,366)	129.59 ± 16.59	129.03 ± 16.4	129.74 ± 16.63	< 0.001
DBP, mmHg, mean ± SD (*n* = 64,366)	81.92 ± 11.17	81.60 ± 11.09	82.00 ± 11.19	< 0.001
ECG abnormality, *n* (%)	14,375 (22.33)	3,056 (22.93)	11,319 (22.17)	0.061

SD, standard deviation; L_Aeq, 8h_, 8-h equivalent A-sound level; FBG, fasting blood glucose; SBP, systolic blood pressure; DBP, diastolic blood pressure; ECG, electrocardiogram. Data were presented as n (%) for categorical variables and mean ± SD for continuous variables.

### 3.2 Relationships of occupational noise exposure with cardiovascular disease risk factors and hearing loss

The associations of occupational noise exposure with FBG, blood pressure, and risk of ECG abnormality and hearing loss were displayed in Table 2. L_Aeq, 8h_ was significantly positively associated with FBG, SBP, and risk of ECG abnormality, HFHL, and SFHL. Each one-unit increment of L_Aeq, 8h_ was related to a 0.006 (95% CI: 0.001–0.011) mmol/L increase in FBG and a 0.036 (95% CI: 0.003–0.068) mmHg increase in SBP. For per-unit increment of L_Aeq, 8h_, there were a 1.4% (95% CI: 0.9%−1.9%), 3.6% (95% CI: 2.9%−4.3%), and 3.7% (95% CI: 3.1%−4.2%) increase in the risk of ECG abnormality, HFHL, and SFHL, respectively. For the categorical analyses, compared to non-exposed participants, high-exposed participants exhibited a 0.115 mmol, 0.803 mmHg, and 0.292 mmHg increment in FBG, SBP, and DBP, respectively; a 33.9 and 29.0% increment in the risk of HFHL and SFHL, respectively (all *P* < 0.05).

**Table 2:** Associations of occupational noise exposure with cardiovascular disease risk factors and hearing loss.

Outcomes	Continuous	Non-exposed	Low-exposed	High-exposed
FBG (*n* = 12,011)	0.006 (0.001, 0.011)	0 (Ref.)	−0.056 (−0.114, 0.001)	0.115 (0.058, 0.172)
SBP (*n* = 49,665)	0.036 (0.003, 0.068)	0 (Ref.)	0.427 (0.065, 0.789)	0.803 (0.441, 1.165)
DBP (*n* = 49,665)	0.012 (-0.010, 0.034)	0 (Ref.)	0.142 (−0.105, 0.389)	0.292 (0.045, 0.538)
ECG abnormality (*n* = 49,668)	1.014 (1.009, 1.019)	1 (Ref.)	0.872 (0.824, 0.923)	1.039 (0.983, 1.099)
High-frequency hearing loss (*n* = 49,668)	1.036 (1.029, 1.043)	1 (Ref.)	1.149 (1.063, 1.242)	1.339 (1.241, 1.447)
Speech-frequency hearing loss (*n* = 49,668)	1.037 (1.031, 1.042)	1 (Ref.)	1.194 (1.128, 1.265)	1.290 (1.218, 1.366)

FBG, fasting blood glucose; SBP, systolic blood pressure; DBP, diastolic blood pressure; ECG, electrocardiogram; Ref., reference. Adjusted for age, gender, height, weight, smoking status, dust exposure, high temperature exposure, hearing protection usage, and length of service.

**Figure 1:**
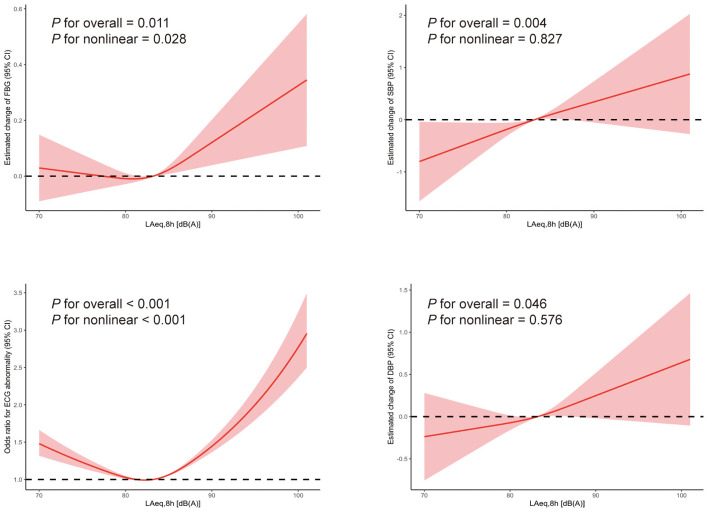
Restricted cubic splines for associations of occupational noise exposure with cardiovascular disease risk factors. L_Aeq, 8h_, 8-h equivalent A-sound level; FBG, fasting blood glucose; SBP, systolic blood pressure; DBP, diastolic blood pressure; ECG, electrocardiogram. Adjusted for age, gender, height, weight, smoking status, dust exposure, high temperature exposure, hearing protection usage, and length of service.

Furthermore, RCS models revealed significant positive linear dose-response association between noise exposure and blood pressure (*P* for overall < 0.05 and *P* for nonlinear >0.05). Meanwhile, we observed a positive non-linear relationship of L_Aeq, 8h_ with FBG and ECG abnormality risk (*P* for overall < 0.05 and *P* for nonlinear < 0.05). FBG levels and the risk of ECG abnormality increased along with increasing L_Aeq, 8h_ when L_Aeq, 8h_ exceeded 80.7 dB(A) and 82.3 dB(A), respectively (Figure 1).

We conducted stratified analyses of the associations between noise exposure and cardiovascular disease risk factors by several characteristics (Table 3). Age modified the relationships of noise exposure with FBG, SBP, and DBP (*P* modification < 0.05), and the effects were stronger in participants >43 years. Dust exposure modified the associations of noise exposure with FBG, DBP, and the risk of ECG abnormality, and the effects were stronger in participants with dust exposure. High temperature exposure modified the relationships of noise exposure with FBG and the risk of ECG abnormality, and the effects were stronger in participants with high temperature exposure. Length of service modified the associations of noise exposure with FBG and SBP, and the effects were stronger in participants with length of service >5 years. The association between noise exposure and hearing loss was modified by age, high temperature exposure, and length of service (Supplementary Table S1).

**Table 3:** Stratified analyses of the associations between occupational noise exposure and cardiovascular disease risk factors.

Stratified characteristics	FBG	SBP	DBP	ECG abnormality
Age				
≤ 43 years	−0.001 (−0.007, 0.004)	−0.023 (−0.064, 0.018)	−0.022 (−0.052, 0.009)	1.010 (1.003, 1.018)
>43 years	0.015 (0.005, 0.024)	0.105 (0.054, 0.155)	0.040 (0.007, 0.072)	1.017 (1.010, 1.024)
*P* modification^a^	**0.003**	**< 0.001**	**0.002**	0.458
Gender				
Male	0.006 (0.001, 0.012)	0.054 (0.019, 0.089)	0.018 (−0.007, 0.042)	1.018 (1.012, 1.023)
Female	0.004 (−0.012, 0.020)	0.050 (−0.039, 0.139)	−0.022 (−0.080, 0.035)	0.992 (0.978, 1.005)
*P* modification^a^	0.785	0.417	0.210	**< 0.001**
BMI				
< 24 kg/m^2^	0.008 (0.001, 0.015)	0.016 (−0.029, 0.061)	−0.014 (−0.045, 0.016)	1.010 (1.003, 1.018)
≥24 kg/m^2^	0.003 (−0.005, 0.011)	0.054 (0.006, 0.101)	0.036 (0.004, 0.068)	1.018 (1.010, 1.025)
*P* modification^a^	0.165	0.441	**0.037**	0.268
Dust exposure				
Yes	0.017 (0.009, 0.025)	0.048 (0.002, 0.094)	0.035 (0.003, 0.066)	1.020 (1.012, 1.027)
No	−0.006 (−0.013, 0.002)	0.022 (−0.024, 0.068)	−0.011 (−0.042, 0.021)	1.008 (1.001, 1.015)
*P* modification^a^	**< 0.001**	0.374	**0.031**	**0.009**
High temperature exposure				
Yes	0.014 (0.007, 0.020)	0.035 (−0.036, 0.105)	0.051 (0.001, 0.101)	1.017 (1.012, 1.023)
No	−0.010 (−0.020, 0.001)	0.039 (0.002, 0.075)	0.006 (−0.018, 0.031)	0.994 (0.981, 1.007)
*P* modification^a^	**< 0.001**	0.531	0.364	**< 0.001**
Length of service				
≤ 5 years	0.001 (−0.007, 0.009)	−0.014 (−0.057, 0.029)	−0.025 (−0.055, 0.004)	1.013 (1.006, 1.020)
>5 years	0.011 (0.003, 0.019)	0.099 (0.049, 0.149)	0.065 (0.031, 0.099)	1.014 (1.007, 1.022)
*P* modification^a^	**< 0.001**	**0.009**	0.065	0.414

BMI, body mass index; FBG, fasting blood glucose; SBP, systolic blood pressure; DBP, diastolic blood pressure; ECG, electrocardiogram. Adjusted for age, gender, height, weight, smoking status, dust exposure, high temperature exposure, hearing protection usage, and length of service. ^a^*P* modification was estimated by adding a product term of stratified characteristic with noise exposure in statistical models. Bold values indicate *P* < 0.05.

### 3.3 Mediating roles of BHFTA and BSFTA in the associations of occupational noise exposure with cardiovascular disease risk factors

**Figure 2:**
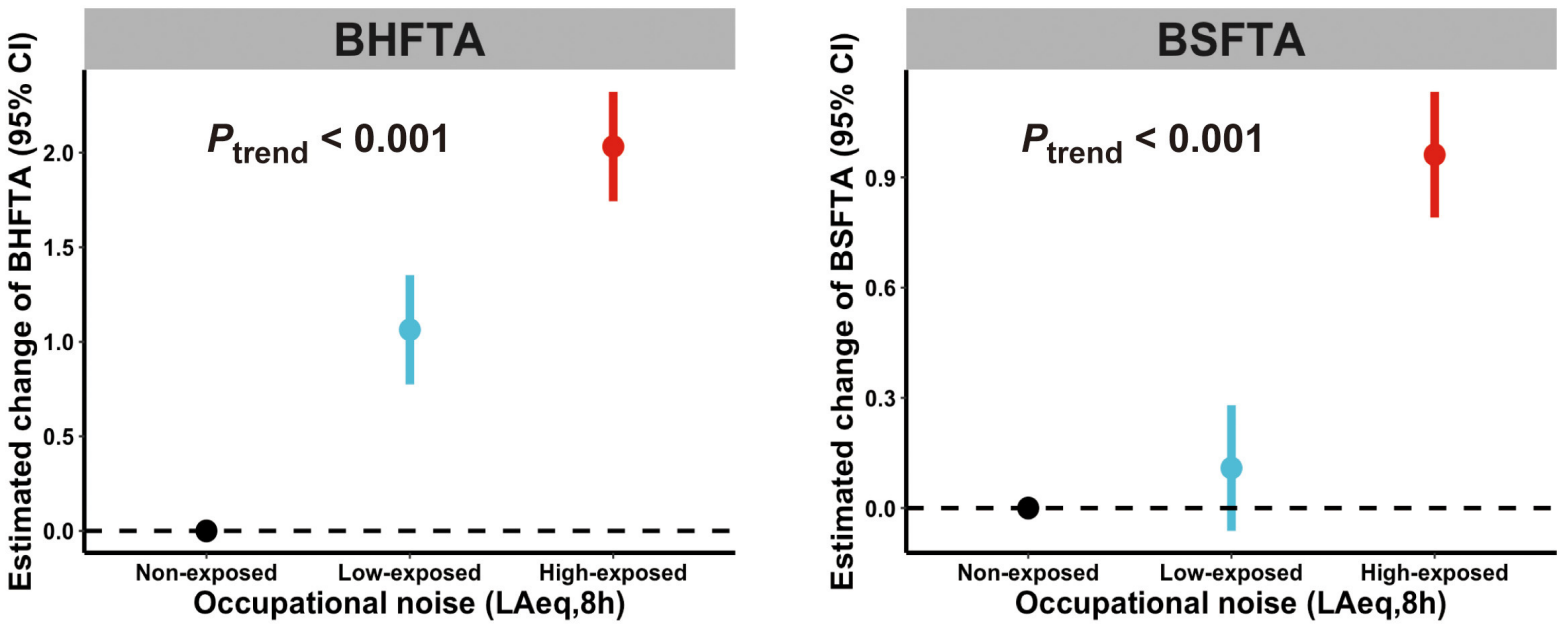
Associations of occupational noise exposure with BHFTA and BSFTA. L_Aeq, 8h_, 8-h equivalent A-sound level; BHFTA, binaural high-frequency threshold on average; BSFTA, binaural speech-frequency threshold on average. Adjusted for age, gender, height, weight, smoking status, dust exposure, high temperature exposure, hearing protection usage, and length of service. *P* for trend was examined through including the median L_Aeq, 8h_ of each noise exposure group as a continuous variable in statistical models.

**Figure 3:**
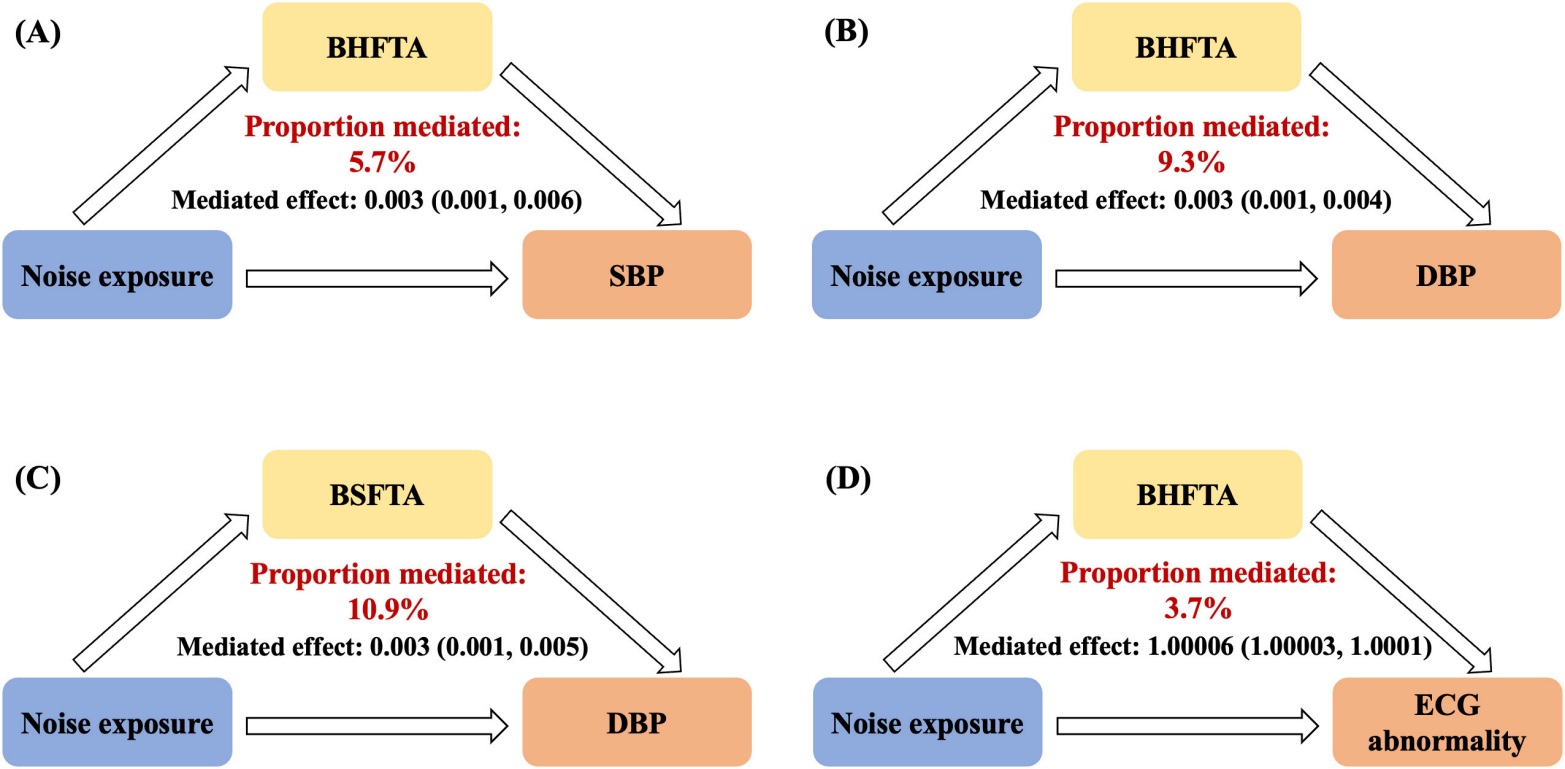
Mediated effects by BHFTA and BSFTA in associations of occupational noise exposure with SBP, DBP, and ECG abnormality. BHFTA, binaural high-frequency threshold on average; BSFTA, binaural speech-frequency threshold on average; FBG, fasting blood glucose; SBP, systolic blood pressure; DBP, diastolic blood pressure; ECG, electrocardiogram. **(A)** mediated effect of BHFTA in noise exposure associated SBP elevation; **(B)** mediated effect of BHFTA in noise exposure associated DBP elevation; **(C)** mediated effect of BSFTA in noise exposure associated DBP elevation; **(D)** mediated effect of BHFTA in noise exposure associated ECG abnormality risk elevation. Adjusted for age, gender, height, weight, smoking status, dust exposure, high temperature exposure, hearing protection usage, and length of service.

As shown in Figure 2, we found significant positive associations of noise exposure with BHFTA and BSFTA (*P* for trend < 0.001). In comparison to non-exposed participants, those with low-exposed and high-exposed of noise manifested a 1.06 (95% CI: 0.77–1.35) dB and 2.03 (95% CI: 1.74–2.32) dB increase in BHFTA, respectively. Compared with non-exposed participants, participants with high-exposed of noise presented 0.96 (95% CI: 0.79–1.13) dB increase in BSFTA. Table 4 demonstrated the relationships of BHFTA and BSFTA with cardiovascular disease risk factors. Fully adjusted models showed that each unit increase of BHFTA was associated with a 0.012 (95% CI: 0.001–0.023) mmHg, 0.010 (95% CI: 0.002–0.017) mmHg, and 0.3% (95% CI: 0.2%−0.5%) increment in SBP, DBP, and the risk of ECG abnormality, respectively. And one-unit increase of BSFTA was related with a 0.019 (95% CI: 0.006–0.031) mmHg increment in DBP. The mediating effects of BHFTA and BSFTA in the associations between noise exposure and cardiovascular disease risk factors were then explored (Figure 3). BHFTA significantly mediated 5.7%, 9.3%, and 3.7% of noise exposure associated elevation in SBP, DBP, and ECG abnormality risk. And BSFTA significantly mediated 10.9% of noise exposure associated elevation in DBP.

**Table 4:** Associations of BHFTA and BSFTA with cardiovascular disease risk factors.

BHFTA/BSFTA	FBG	SBP	DBP	ECG abnormality
BHFTA				
Model 1	**0.003 (0.001, 0.004)**	**0.072 (0.062, 0.082)**	**0.037 (0.030, 0.044)**	**1.004 (1.003, 1.005)**
Model 2	−0.001 (−0.002, 0.002)	**0.015 (0.004, 0.026)**	**0.012 (0.004, 0.019)**	**1.004 (1.002, 1.005)**
Model 3	−0.001 (−0.003, 0.001)	**0.012 (0.001, 0.023)**	**0.010 (0.002, 0.017)**	**1.003 (1.002, 1.005)**
BSFTA				
Model 1	0.002 (−0.002, 0.005)	**0.069 (0.052, 0.086)**	**0.044 (0.033, 0.056)**	1.001 (0.998, 1.004)
Model 2	−0.001 (−0.004, 0.003)	0.008 (−0.010, 0.027)	**0.023 (0.010, 0.036)**	0.998 (0.995, 1.001)
Model 3	−0.001 (−0.004, 0.003)	0.002 (−0.017, 0.020)	**0.019 (0.006, 0.031)**	0.998 (0.995, 1.001)

BHFTA, binaural high-frequency threshold on average; BSFTA, binaural speech-frequency threshold on average; FBG, fasting blood glucose; SBP, systolic blood pressure; DBP, diastolic blood pressure; ECG, electrocardiogram. Model 1: crude model. Model 2: adjusted for age, gender, height, weight, smoking status. Model 3: adjusted for age, gender, height, weight, smoking status, dust exposure, high temperature exposure, hearing protection usage, and length of service. Bold values indicate *P* < 0.05.

## 4 Discussion

In this study, we systematically discovered that increased noise exposure was related with elevated risk of hearing loss, FBG level, blood pressure level, and risk of ECG abnormality in a large occupational population. The adverse effects were more obvious in participants >43 years, workers with dust or high temperature exposure, and workers with length of service >5 years. Importantly, mediation analyses identified that BHFTA and BSFTA partially mediated the associations of occupational noise exposure with blood pressure level and risk of ECG abnormality. These findings demonstrate significant associations between occupational noise exposure and cardiovascular disease risk factors, which partially mediated by hearing loss, emphasizing the public health issues about the harmful effects of occupational noise exposure on cardiovascular health and the mediating role of hearing loss.

Our results provided compelling evidence that noise exposure could increase cardiovascular disease risk among the occupational population. An increasing number of studies have explored the association between noise exposure and changes of FBG among adults; however, the results were inconsistent. Based on over 27,000 participants from a natural cohort in Shanghai, a study observed that environmental noise exposure was related with higher FBG (12). In contrast, another study conducted among 854 Chinese adults did not find significant association of occupational noise exposure with elevated glucose (24). A meta-analysis included 44,698 individuals demonstrated that higher level of occupational noise exposure may lead to a greater risk of metabolic syndrome when compared to traffic noise exposure (25). Our results add to evidence on the possible association between noise exposure and elevated FBG in the Chinese occupational population. Moreover, we observed this association was non-linear and FBG levels significantly increased when L_Aeq, 8h_ exceeded 80.7 dB(A), indicating the current occupational noise standard of 85 dB(A) in China may be insufficient to prevent hyperglycemia. Our findings underscored the importance of mitigating high-intensity occupational noise pollution to protect health on glucose metabolism.

Our study revealed that noise exposure was positively associated with blood pressure. Previous studies reported similar results in workers with occupational noise exposure. Chen et al. found that 1,390 workers exposed to occupational noise had significantly higher SBP, DBP, and risk of hypertension than 1,399 matched controls from the non-exposed general population in China (26). Consistently, a study performed in the United States discovered that participants chronically exposed to occupational noise had increased prevalence of diastolic hypertension compared to never exposed subjects (27). Similar results were reported in studies concerning road traffic noise, where Foraster et al. documented the association of long-term exposure to indoor traffic noise with increased SBP and hypertension (28). An animal experiment also demonstrated increased blood pressure and oxidative stress in mouse models of diabetes after 4 days of aircraft noise exposure (29). Besides, we found that age, BMI, dust exposure, and length of service modified the association of occupational noise exposure with blood pressure, and the detrimental effects were more obvious in participants >43 years, overweight people, workers with dust exposure, and workers with length of service >5 years.

We found significant association between noise exposure and risk of ECG abnormality with fully adjustment, and the harmful effect of high-intensity noise exposure was highlighted. While studies about the effect of noise exposure on ECG remain scarce, a meta-analysis study reported that the risk of developing ECG abnormality among workers exposed to noise is 2.27 times higher than control groups (30). In a recent experiment of three male macaque monkeys, one monkey showed an RS-type QRS wave and inverted T waves after noise exposure of 90 dB pure tone, though the other two monkeys exhibited no significant changes in ECG parameters (31). Future epidemiological and experimental studies are warranted to investigate the effect of noise exposure on ECG.

In addition to occupational noise, occupational population are frequently exposed to multiple occupational hazardous factors, among which dust exposure and high temperature exposure are the most prevalent (32, 33). In the present study, we found the associations between occupational noise exposure and cardiovascular risk factors were modified by dust exposure and high temperature exposure, and the harmful effects were more apparent among subgroups with dust exposure or high temperature exposure. A nested case-control study also suggested that exposure to noise, dust, and high temperature elevates the hypertension risk of steelworkers, and the risk of hypertension is more obvious among workers with both dust and noise exposure (34). Our findings highlight that more attention should be paid to the prevention of noise-related cardiovascular effects among workers with co-exposure to dust and high temperature.

The possible mechanisms underlying the link between noise exposure and cardiovascular disease risk factors are largely uncharted. It is hypothesized that chronic exposure to noise may activate the autonomic and endocrine systems, triggering a cascade of stress hormones that lead to adverse changes in blood glucose, blood pressure, and ECG patterns (35). Herein, we illustrate the mediating roles of BHFTA and BSFTA in the relationships of noise exposure with cardiovascular disease risk factors, suggesting that hearing loss may play an important role in associating noise exposure with cardiovascular disease risk. An earlier 10-year prospective study observed significant noise exposure-related elevations in hearing threshold level among 456 construction workers (36), which is consistent with our finding about positive associations of noise exposure with BHFTA and BSFTA. Toxicologic studies also supported hearing loss induced by noise exposure in mice (37, 38). Meanwhile, we observed positive associations of BHFTA and BSFTA with blood pressure. Similar with our results, another large epidemiological research demonstrated that bilateral hearing loss was related with a higher risk of hypertension, no matter for speech-frequency or high-frequency hearing loss (39). Mechanistically, noise-related hearing loss may lead to calcium overload and elevated production of reactive oxygen species, thereby triggering oxidative stress that contributes to the progression of cardiovascular disease, including increased blood pressure and risk of ECG abnormality (40). The mediation results should be interpreted as exploratory with caution, as they are based solely on statistical mediation analysis. More toxicological researches are highly encouraged to explore the latent mechanisms.

Our research has several advantages. Firstly, among a large-scale sample size over 60,000 occupational workers with noise exposure, we observed the powerful relationships between increased noise exposure and cardiovascular disease risk factors. Secondly, we further utilized mediation analyses to find the latent role of hearing loss in the adverse effects of noise exposure on cardiovascular disease risk factors, providing primary evidence for more deep mechanistic studies in the future. Thirdly, we observed non-linear associations between noise exposure and FBG levels as well as risk of ECG abnormality, with L_Aeq, 8h_ exceeded around 82 dB(A) identified as a potential turning point, indicating the current occupational noise standard of 85 dB(A) in China may be insufficient to prevent detrimental cardiovascular effects. However, because this threshold was identified in the present observational study, it warrants validation in additional occupational population datasets. Nonetheless, some limitations of this research should be noted. First, the evidence of causal relationships is not persuasive enough due to the observational nature of cross-sectional design. Further prospective study is warranted to confirm our results. Second, the exploration of mediating mechanism was conducted solely through statistical mediation analysis models. Thus, further toxicological experiments are needed to provide deeper mechanistic insights. Third, possible exposure misclassification in occupational noise exposure may be introduced by using enterprise-level average noise measurements, which might potentially attenuate or obscure the true exposure-response relationships. Workers within the same enterprise may experience heterogeneous noise exposures depending on workstation, job task, rotation patterns, etc. Nevertheless, given the large-scale of this occupational epidemiological study, enterprise-level average noise measurements represented a practical and feasible approach for exposure assessment. Finally, caution should be exercised when generalizing the findings to other populations, as this study was performed among an occupational population.

## 5 Conclusions

Overall, this epidemiological study revealed significant positive associations between noise exposure and cardiovascular disease risk factors among a large-scale occupational population, which were more pronounced in older subjects, workers with dust exposure or high temperature exposure, and workers with length of service >5 years. Moreover, the associations of occupational noise with blood pressure and risk of ECG abnormality were mediated in part by hearing loss. Our findings highlight more attentions should be paid on high-intensity occupational noise associated adverse cardiovascular effects, particularly among older workers, those exposed to dust or high temperature, and those with longer working duration. Efficient strategies for reducing occupational noise exposure, including limiting industrial emissions and high-quality personal protective equipment, should be enhanced to control the public health threat from occupational noise pollution. More researches are urged to affirm these discoveries.

## Data Availability

The original contributions presented in the study are included in the article/Supplementary material, further inquiries can be directed to the corresponding authors.
