# Supplementary material for: Occupational noise exposure and cardiovascular disease risk factors in China: mediating role of hearing loss
Source: Front Public Health. 2026 Aug 4;14:1901898. doi: 10.3389/fpubh.2026.1901898 (PMC13481372; doi:10.3389/fpubh.2026.1901898)
Supplement: Supplementary file 1 [file Table_5.docx]

**Supplementary Material**

**Table S1.** Stratified analyses of the associations between occupational noise exposure and hearing loss

| **Stratified characteristics** | **High-frequency hearing loss** | **Speech-frequency hearing loss** |
| --- | --- | --- |
| Age |  |  |
| ≤43 years | 1.043 (1.030, 1.055) | 1.048 (1.040, 1.057) |
| >43 years | 1.029 (1.021, 1.038) | 1.027 (1.020, 1.033) |
| *P* modification ^a^ | **0.023** | **<0.001** |
| Gender |  |  |
| Male | 1.036 (1.029, 1.043) | 1.038 (1.032, 1.043) |
| Female | 1.028 (1.003, 1.054) | 1.028 (1.013, 1.043) |
| *P* modification ^a^ | 0.569 | 0.252 |
| BMI |  |  |
| <24 kg/m^2^ | 1.033 (1.023, 1.043) | 1.035 (1.028, 1.043) |
| ≥24 kg/m^2^ | 1.038 (1.029, 1.048) | 1.038 (1.031, 1.046) |
| *P* modification ^a^ | 0.178 | 0.408 |
| Dust exposure |  |  |
| Yes | 1.022 (1.013, 1.032) | 1.034 (1.027, 1.041) |
| No | 1.048 (1.037, 1.058) | 1.037 (1.030, 1.045) |
| *P* modification ^a^ | **0.003** | 0.689 |
| High temperature exposure |  |  |
| Yes | 1.087 (1.066, 1.108) | 1.095 (1.080, 1.111) |
| No | 1.027 (1.020, 1.035) | 1.027 (1.022, 1.033) |
| *P* modification ^a^ | **<0.001** | **<0.001** |
| Length of service |  |  |
| ≤5 years | 1.018 (1.008, 1.028) | 1.033 (1.026, 1.040) |
| >5 years | 1.053 (1.043, 1.063) | 1.041 (1.033, 1.049) |
| *P* modification ^a^ | **<0.001** | **0.007** |

Abbreviations: BMI, body mass index.

Adjusted for age, sex, height, weight, smoking status, dust exposure, high temperature exposure, hearing protection usage, and length of service.

^a^ *P* modification was estimated by adding a product term of stratified characteristic with noise exposure in statistical models.
